# Expanded dengue syndrome presenting with acute liver failure, acute kidney injury, pancreatic involvement, coagulopathy, and multiple intracranial hemorrhages in a young child: a case report

**DOI:** 10.1186/s13256-022-03348-0

**Published:** 2022-03-29

**Authors:** V. Thadchanamoorthy, Kavinda Dayasiri

**Affiliations:** 1 Faculty of Health Care Sciences, Eastern University, Chenkaladi, Sri Lanka; 2 Department of Paediatrics, Faculty of Medicine, University of Kelaniya, Colombo, Sri Lanka

**Keywords:** Dengue hemorrhagic fever, Intracranial hemorrhage, Multiorgan, Arthritis

## Abstract

**Background:**

Dengue is a mosquito-borne viral infection that typically occurs in tropical and subtropical countries. The clinical manifestations of dengue infection range from an asymptomatic subclinical course to severe dengue shock syndrome. Besides, dengue can affect any organ in the body and can present with atypical manifestations.

**Case presentation:**

We report a 6-year-old previously healthy Tamil child who had dengue complicated with multiorgan involvement. She initially presented with high fever, headache, body aches for 5 days, blood and mucus diarrhea, hematuria, and right knee joint swelling for 2 days. Dengue NS1 antigen was positive on day 2 of febrile illness. She was managed symptomatically in the local hospital for 3 days and transferred to the tertiary care hospital for further management. She was eventually diagnosed as having dengue hemorrhagic fever complicated with multiorgan involvement including acute liver failure, pancreatic involvement, coagulopathy, arthritis, acute kidney injury, and multiple intracranial hemorrhages. The constellation of disease manifestations was identified as expanded dengue syndrome. She was managed with fresh blood, platelet, and cryoprecipitate transfusions and intravenous antibiotics in addition to renal and liver support in the intensive care unit. On day 14 of illness, she deteriorated while on the ventilator and died due to multiple intracranial hemorrhages.

**Conclusion:**

The reported child with dengue hemorrhagic fever developed several unusual presentations such as acute liver and renal failure, disseminated intravascular coagulopathy, pancreatic involvement, and multiple intracranial hemorrhages, which form part of expanded dengue syndrome. In the seriously unwell child, it is important to look for unusual complications actively to improve outcomes.

## Introduction

Dengue infection is a mosquito-borne, neglected tropical disease. Dengue is a serious health problem globally, and its incidence has been on the rise over recent years all over the world [1, 2]. Epidemics of dengue have occurred every few years from 1991 to date in Sri Lanka [2–4] with the highest number of suspected cases being reported in 2017 [4]. Dengue fever (DF) and dengue hemorrhagic fever (DHF) are caused by four antigenitically distinct dengue virus subtypes. Dengue virus is transmitted by *Aedes aegypti* [5]. After an incubation period of 4–10 days, the mosquitoes transmit the virus for the rest of their life; the infection varies from self-limiting subclinical infection to lethal complications such as dengue shock syndrome and multiorgan failure [5]. There are several atypical or uncommon presentations of dengue infection, including hepatitis, encephalitis, myocarditis, pancreatitis, and arthritis [6]. Expanded dengue syndrome incorporates a wide spectrum of uncommon presentations of this common disease. The World Health Organization (WHO) established guidelines for expanded dengue syndrome in 2012, and subsequently, several pediatric cases of expanded dengue syndrome were reported, more in association with DHF than DF. Unusual presentations could be associated with coinfection, comorbidities, and lethal complications following prolonged shock [6]. Early diagnosis might help to prevent unfavorable outcomes. In addition, patients should also seek medical attention without delay to prevent severe complications. Unusual presentations of expanded dengue syndrome are reported less frequently in children as compared with adults. We present the case of a young girl who presented with expanded dengue syndrome and died due to multiple intracranial hemorrhages.

## Case history

A 6-year-old previously health Tamil girl was transferred to the tertiary hospital with continuous high fever (highest 41.8 °C), severe headache, body ache for 5 days, blood and mucus diarrhea, hematuria, and right knee joint swelling for 2 days duration. She was admitted to local hospital with positive NS1 antigen. Complete blood count on admission revealed white blood count of 5.2 × 10^3^/mm^3^, hemoglobin of 11 g/dl, and platelet count of 150 × 10^3/^ cumm. She was managed symptomatically with monitoring of serial blood counts. As she developed gross hematuria and one episode of blood and mucus diarrhea, she was transferred to the tertiary care hospital for further management. Her urine output was 0.4 ml/kg/h. No other bleeding manifestations were reported by the child. Her paracetomol intake was appropriate for the weight. There was no past history of medical illness, surgical intervention, or hospitalization of the child. The child’s father also had dengue DHF 2 weeks before but recovered within 2 weeks. She had age-appropriate development and immunization. Both parents and their relatives were worried about the child’s condition on admission as this was the only child of this family.

Physical examination revealed that she was overweight (33 kg), ill, irritable, febrile, and mildly icteric, and had bilateral congested conjunctiva and mild bleeding from gums. Her vital signs on admission were pulse rate of 152/minute with low volume, blood pressure of 140/120 mmHg, respiratory rate of 32/minute, capillary refilling time of 3 seconds, and cold and clammy extremities. Abdominal examination revealed 5 cm hepatomegaly and 3 cm splenomegaly with generalized abdominal tenderness. Respiratory system examination revealed reduced air entry on the right side with clear lungs. She had right knee joint swelling with limited movements. In-ward ultrasound revealed hepatosplenomegaly with free fluids in the pericolic region and ascites. Provisional diagnosis of DHF with early shock was made, and she was resuscitated with fluids and supportive management. She was transferred to the intensive care unit (ICU), and further resuscitation was done with dextran and blood transfusion. The child was commenced on intravenous fluids at 7 ml/kg/h. Her urine output was 0.5 ml/kg/h. Arterial blood gas showed metabolic acidosis (pH 7.28, HCO_3_ 12 mmol/L), which was subsequently corrected with further intravenous fluids and bicarbonate infusion.

Investigations at the tertiary care hospital revealed leukocytosis with neutrophil predominance (WBC: 15 × 10^3^/cumm, N: 80%, Hb: 15 g/dl. platelets: 95 × 10^3^/cumm, PCV: 46%), and high C-reactive protein (CRP: 96 mg/dl). Urine microscopy showed field full of red cells with more than 10 pus cells per high-power field. The results of other investigations are presented in Table 1. Since the child had raised inflammatory markers, she was started on intravenous cefotaxime as for secondary bacterial infection while awaiting blood and urine cultures.

**Table 1:** Investigations in intensive care unit

Investigation	Value on day 2 of ICU	Value on day 5 of ICU	Reference range
WBC	10 × 10^3^	3.5 × 10^3^	4–10 × 10^3^ cumm
Neutrophil	3.5 × 10^3^	1 × 10^3^	
Lymphocytes	4.5 × 10^3^	2.1 × 10^3^	
Hematocrit	46	35	32–35
Hemoglobin	15 g/dL	13 g/dL	13–14 g/dL
Platelets	60 × 10^3^	108 g/dL	150–450/dL
ESR	10/hour	20/hour	<20/first hour
Blood picture	No abnormal cells, thrombocytopenia		
Antinuclear antibody		Negative	
Complement (C_3_,C_4_)		Negative	
Serum albumin	28 g/l	24	34–50 g/L
SGOT	1260 U/L	534 U/L	10–40 U/L
SGPT	745 U/L	3245 U/L	10–40 U/L
Random blood sugar	98 mg/dL	40 mg/dl	80–120 mg/dL
Serum bilirubin total	64 µmol/L	124 µmol/L	3–20 µmol/L
Serum bilirubin direct	34 µmol/L	74 µmol/L	< 3 µmol/L
Prothrombin time	24 seconds	45 seconds	10–14 seconds
INR	3.1	4	2–2.2
APTT	34 seconds	60 seconds	25–35 seconds
Serum fibrinogen	98 mg/dL		200–400 mg/dL
D-dimer		Less than 0.25	< 0.5
Serum triglycerides	184 mg/dL		< 150 mg/dL
Serum LDH		586 U/L	140–280 U/L
Serum sodium	134 meq/L	141 meq/L	135–145 meq/L
Serum potassium	5.1 meq/L	5.8 meq/L	3.5–5.1 meq/L
Blood urea	60 mg/dL	80 mg/dL	
Serum creatinine	0.8 mg/dL	1.8 mg/dL	0.5–1.2 mg/dL
Serum ferritin	325 μg/L	360 μg/L	24–336 μg/L
Serum amylase	–	220 U/L	30–110 U/L
Serum lipase	–	86 U/L	0–160 U/L

ICU: Intensive Care Unit

On the third day in ICU, she developed a generalized tonic and clonic seizure and deteriorated on Glasgow Coma Scale and needed ventilatory care. Computerized tomography of brain at this point was normal, but her blood pressure, pulse and pulse volume, and capillary refilling time were normal after 48 hours of intensive care management. She continued to have high liver transaminases and serum bilirubin with evidence of acute liver failure. Her amylase was high and indicated pancreatic involvement. She also had bleeding from puncture sites suggesting coagulopathy, subsequently confirmed by deranged coagulation profile. She was resuscitated with blood and blood products including platelets, fresh frozen plasma, and cryoprecipitate to control bleeding. Serum ferritin, fibrinogens, lipid profile, and bone marrow examination were normal, and hemophagocytic lymphocytic histiocytosis (HLH) was excluded.

Although urine culture was negative, blood culture grew *Klebsiella* sensitive to cefotaxime. Her fever settled gradually while on intravenous antibiotics. However, her clinical condition deteriorated with evidence of deteriorating renal function, and she developed recurrent hypoglycemic convulsions subsequently. She was started on renal replacement therapy and liver support therapy. Ultimately she died while receiving ventilatory care due to multiple intracranial hemorrhages on day 14 of illness.

## Discussion

Although there are a number of reports of expanded dengue syndrome in adults [7], this entity is rarely documented in children younger than 12 years. Gastrohepatic manifestations and acute pancreatitis are among the more commonly reported atypical manifestations in adults [7] as compared with neurological manifestations and acute kidney injury. Intracranial hemorrhage has been rarely reported even among adults [8–10] and carried high mortality [11]. Similar to the reported child, intracerebral hemorrhage has occurred in many reported adult patients when platelets are above 20,000/cumm [11]. This fact highlights the importance of urgent neuroimaging in those with deteriorating consciousness despite higher platelet counts. The presentation in this child was unusual in that several factors such as acute liver failure, septicemia, and uremia contributed to deteriorating consciousness in addition to intracerebral hemorrhages.

Disseminated intravascular coagulation was reported in approximately 1.5% of children with confirmed dengue fever in one series [12]. Most of the bleeding manifestations comprised bleeding manifestations of skin, melena, gum bleeding, and epistaxis. Only two children with intracranial hemorrhages have been reported, but none reported to have hematuria [12]. Splenomegaly was another unusual finding of expanded dengue and has been reported more commonly in children, similar to this patient [13]. Serum amylase too was elevated in the child, suggesting evidence of pancreatic involvement. Acute pancreatitis is also rarely documented in literature [14]. Although the exact etiology is unknown, direct viral invasion, ischemia due to dengue shock, and autoimmune-mediated damage are proposed mechanisms [15].

The WHO introduced the spectrum of expanded dengue syndrome in 2012 and included atypical and unusual manifestations of neurological, renal, hepatic, or other isolated organ systems, which occurred as complications of severe profound shock or coinfections or secondary to underlying host conditions/diseases [6]. Seizures, intracranial hemorrhages, pancreatitis, acute hepatitis and hepatic failure, and acute kidney injury reported in this child form part of the spectrum of expanded dengue syndrome. Acute splenic rupture is also reported as an unusual manifestation [16]. However, in this child, only splenomegaly was noted, with no evidence of splenic rupture.

End-organ dysfunction in expanded dengue syndrome is most commonly caused by dengue shock and bleeding [17]. This child had the whole critical phase managed in a hospital setting; however, it was likely that the hemodynamic instability arising from severe dengue and septicemia both contributed to multiorgan dysfunction despite timely interventions, supportive care, and fluid management upon admission to the tertiary care unit. It is recommended that blood transfusions be used early in dengue shock to mitigate the effects of tissue hypoxia and risk of further and more severe bleeding manifestations [17]. Oliguric renal failure was managed in this child with renal replacement therapy given the potential harmful effects of fluid challenge on the risk of fluid overload and related complications in this child such as raised intracranial pressure and brain edema. Although neutrophil-predominant leukocytosis together with high fever can be observed in dengue shock, positive blood cultures also supported *Klebsiella* septicemia necessitating management of both severe dengue and *Klebsiella* septicemia in this child.

Overall, the management of this patient was challenging from the onset of dengue fever. The disease followed an unusual path with several atypical manifestations such as hematuria, acute liver and renal failure, disseminated intravascular coagulopathy, pancreatic involvement, multiple intracerebral hemorrhages, and splenomegaly. The management was further complicated by concurrent *Klebsiella* septicemia. The case highlights the importance of early and careful fluid management during the critical phase, early administration of blood transfusions in the presence of shock and bleeding, and careful monitoring for unusual manifestations.

As this was the only child in the whole family of these young parents, they were worried and disturbed psychologically. We subsequently arranged several counseling sessions with the family for 1 year with the external multidisciplinary team. The parents recovered slowly with the hope that their child reached heaven.

## Conclusion

The reported child with dengue hemorrhagic fever developed several unusual presentations such as acute liver and renal failure, disseminated intravascular coagulopathy, pancreatic involvement, and multiple intracranial hemorrhages, which form part of expanded dengue syndrome. It is crucial that the onset of critical phase is detected accurately and in a timely fashion by meticulous monitoring. Careful fluid management during the critical phase and early administration of blood transfusions in the presence of shock and bleeding are important to prevent damage to multiple end organs. In the seriously unwell child, it is important to look for unusual complications actively to improve outcomes.

## Data Availability

The data that support the findings of this case report are available from the Medical Records Department, Batticaloa Teaching Hospital, but restrictions apply to the availability of these data, which were used under license for the current report and so are not publicly available. Data are, however, available from the authors upon reasonable request and with permission of Medical Records Department, Batticaloa Teaching Hospital, Sri Lanka.

## Abbreviations

DF: Dengue fever
DHF: Dengue hemorrhagic fever
WHO: World Health Organization
HLH: Hemophagocytic lymphocytic histiocytosis

## References

[1] Gubler DJ. Dengue and dengue hemorrhagic fever. Clin Microbiol Rev. 1998;11(3):480–96. 10.1128/cmr.11.3.480 PMC88892 9665979

[2] Kulatilaka TA, Jayakuru WS. Control of dengue/dengue haemorrhagic fever in Sri Lanka. Dengue Bull. 1998;22:53–61.

[3] Epidemiology Unit, Ministry of Health, Sri Lanka. Distribution of notification (H399) dengue cases by month. http://www.epid.gov.lk/web/index.php?option=com_casesanddeaths&Itemid=448&lang=en#. Accessed 15 May 2018.

[4] Tissera HA, Jayamanne BDW, Raut R, *et al*. Severe dengue epidemic, Sri Lanka, 2017. Emerg Infect Dis. 2020;26(4):682–91. 10.3201/eid2604.190435. PMC7101108 32186490

[5] World health organization. Fact sheet: dengue and severe dengue. https://www.who.int/newsroom/fact-sheets/detail/dengue-and-severe-dengue.

[6] World Health organization Comprehensive guidelines for prevention and treatment of dengue and dengue hemorrhagic fever. New Delhi: WHO, SEARO; revised and expanded edition.

[7] Mohanty B, Sunder A, Pathak S. Clinicolaboratory profile of expanded dengue syndrome—our experience in a teaching hospital. J Fam Med Prim Care. 2019;8(3):1022–7. 10.4103/jfmpc.jfmpc_12_19. PMC6482768 31041245

[8] Ahmed S, Ali N, Tariq WU. Neurological manifestations as presenting feature in dengue fever. J Coll Phys Surg Pak. 2007;17(4):236–7. 17462188

[9] Jayasinghe NS, Thalagala E, Wattegama M, *et al*. Dengue fever with diffuse cerebral hemorrhages, subdural hematoma and cranial diabetes insipidus. BMC Res Notes. 2016;9:265. 10.1186/s13104-016-2068-5 PMC4862122 27165581

[10] Sam JE, Gee TS, Nasser AW. Deadly intracranial bleed in patients with dengue fever: a series of nine patients and review of literature. J Neurosci Rural Pract. 2016;7(3):423–34. 10.4103/0976-3147.182777 PMC4898113 27365962

[11] Rajapakse S, Wattegama M, Weeratunga P, Sigera PC, Fernando SD. Beyond thrombocytopaenia, haemorrhage and shock: the expanded dengue syndrome. Pathog Glob Health. 2018;112(8):404–14. 10.1080/20477724.2018.1552645. PMC6327626 30507366

[12] Pothapregada S, Kamalakannan B, Thulasingham M, Sampath S. Clinically profiling pediatric patients with dengue. J Glob Infect Dis. 2016;8(3):115–20. 10.4103/0974-777X.188596. PMC4997795 27621562

[13] Jhamb R, Kumar A, Ranga GS, Rathi N. Unusual manifestations in dengue outbreak 2009, Delhi, India. J Commun Dis. 2010;42(4):255–61. 22471194

[14] Jain V, Gupta O, Rao T, Rao S. Acute pancreatitis complicating severe dengue. J Glob Infect Dis. 2014;6(2):76–8. 10.4103/0974-777X.132050. PMC4049044 24926168

[15] Wijekoon CN, Wijekoon PW. Dengue hemorrhagic fever presenting with acute pancreatitis. Southeast Asian J Trop Med Public Health. 2010;41:864–6. 21073060

[16] Gulati S, Maheshwari A. Atypical manifestations of dengue. Trop Med Int Health. 2007;12(9):1087–95. 10.1111/j.1365-3156.2007.01891.x. 17875019

[17] Kalayanarooj S, Rothman AL, Srikiatkhachorn A. Case management of dengue: lessons learned. J Infect Dis. 2017;215(suppl_2):S79–88. 10.1093/infdis/jiw609. PMC5853291 28403440

